# Active prophages in coral-associated *Halomonas* capable of lateral transduction

**DOI:** 10.1093/ismejo/wrae085

**Published:** 2024-05-13

**Authors:** Ziyao Liu, Kaihao Tang, Yiqing Zhou, Tianlang Liu, Yunxue Guo, Duoting Wu, Xiaoxue Wang

**Affiliations:** Key Laboratory of Tropical Marine Bio-resources and Ecology, Guangdong Key Laboratory of Marine Materia Medica, Innovation Academy of South China Sea Ecology and Environmental Engineering, South China Sea Institute of Oceanology, Chinese Academy of Sciences, 164 West Xingang Road, Guangzhou 510301, China; University of Chinese Academy of Sciences, No.1, Yanqihu East Road, Huairou District, Beijing 101408, China; Key Laboratory of Tropical Marine Bio-resources and Ecology, Guangdong Key Laboratory of Marine Materia Medica, Innovation Academy of South China Sea Ecology and Environmental Engineering, South China Sea Institute of Oceanology, Chinese Academy of Sciences, 164 West Xingang Road, Guangzhou 510301, China; University of Chinese Academy of Sciences, No.1, Yanqihu East Road, Huairou District, Beijing 101408, China; Southern Marine Science and Engineering Guangdong Laboratory (Guangzhou), No.1119, Haibin Road, Nansha District, Guangzhou 511458, China; Key Laboratory of Tropical Marine Bio-resources and Ecology, Guangdong Key Laboratory of Marine Materia Medica, Innovation Academy of South China Sea Ecology and Environmental Engineering, South China Sea Institute of Oceanology, Chinese Academy of Sciences, 164 West Xingang Road, Guangzhou 510301, China; Key Laboratory of Tropical Marine Bio-resources and Ecology, Guangdong Key Laboratory of Marine Materia Medica, Innovation Academy of South China Sea Ecology and Environmental Engineering, South China Sea Institute of Oceanology, Chinese Academy of Sciences, 164 West Xingang Road, Guangzhou 510301, China; University of Chinese Academy of Sciences, No.1, Yanqihu East Road, Huairou District, Beijing 101408, China; Key Laboratory of Tropical Marine Bio-resources and Ecology, Guangdong Key Laboratory of Marine Materia Medica, Innovation Academy of South China Sea Ecology and Environmental Engineering, South China Sea Institute of Oceanology, Chinese Academy of Sciences, 164 West Xingang Road, Guangzhou 510301, China; University of Chinese Academy of Sciences, No.1, Yanqihu East Road, Huairou District, Beijing 101408, China; Southern Marine Science and Engineering Guangdong Laboratory (Guangzhou), No.1119, Haibin Road, Nansha District, Guangzhou 511458, China; Key Laboratory of Tropical Marine Bio-resources and Ecology, Guangdong Key Laboratory of Marine Materia Medica, Innovation Academy of South China Sea Ecology and Environmental Engineering, South China Sea Institute of Oceanology, Chinese Academy of Sciences, 164 West Xingang Road, Guangzhou 510301, China; Key Laboratory of Tropical Marine Bio-resources and Ecology, Guangdong Key Laboratory of Marine Materia Medica, Innovation Academy of South China Sea Ecology and Environmental Engineering, South China Sea Institute of Oceanology, Chinese Academy of Sciences, 164 West Xingang Road, Guangzhou 510301, China; University of Chinese Academy of Sciences, No.1, Yanqihu East Road, Huairou District, Beijing 101408, China; Southern Marine Science and Engineering Guangdong Laboratory (Guangzhou), No.1119, Haibin Road, Nansha District, Guangzhou 511458, China

**Keywords:** prophage, Halomonas, phage, coral microbiome, temperate phage, lateral transduction

## Abstract

Temperate phages can interact with bacterial hosts through lytic and lysogenic cycles via different mechanisms. Lysogeny has been identified as the major form of bacteria–phage interaction in the coral-associated microbiome. However, the lysogenic-to-lytic switch of temperate phages in ecologically important coral-associated bacteria and its ecological impact have not been extensively investigated. By studying the prophages in coral-associated *Halomonas meridiana*, we found that two prophages, Phm1 and Phm3, are inducible by the DNA-damaging agent mitomycin C and that Phm3 is spontaneously activated under normal cultivation conditions. Furthermore, Phm3 undergoes an atypical lytic pathway that can amplify and package adjacent host DNA, potentially resulting in lateral transduction. The induction of Phm3 triggered a process of cell lysis accompanied by the formation of outer membrane vesicles (OMVs) and Phm3 attached to OMVs. This unique cell-lysis process was controlled by a four-gene lytic module within Phm3. Further analysis of the Tara Ocean dataset revealed that Phm3 represents a new group of temperate phages that are widely distributed and transcriptionally active in the ocean. Therefore, the combination of lateral transduction mediated by temperate phages and OMV transmission offers a versatile strategy for host–phage coevolution in marine ecosystems.

## Introduction

Viruses represent the most abundant biological entities in the ocean [1]. Most marine viruses are bacteriophages that infect bacteria and archaea and play vital roles in influencing the community composition, biomass, and genetic diversity of their hosts [2–5]. Bacteriophages infect hosts through two major life strategies: lytic and lysogenic infection cycles [6]. Temperate (lysogenic) phages are viruses that follow an alternative life cycle and can integrate into the host genome and become prophages. Prophages are generally abundant in bacterial genomes [7] and are major drivers of genetic plasticity [8]. Approximately 28%–71% of marine bacterial isolates contain prophages [9]. The paradigm regarding the choice between the phage lytic and lysogenic pathways has been well studied for the lambda (λ) phage and the *Escherichia coli* host. Upon entering the *E. coli* host, a “parasitic” temperate phage such as λ integrates its genome into the host genome and immediately enters the lytic cycle when mitomycin C (MMC) treatment elicits the SOS response in the lysogen [10]. In contrast, many other prophages maintain a lysogenic state during host DNA damage and face the same “life or death” fate as the host. These prophages can establish a “mutualistic” relationship with their hosts and only choose to become active when they receive specific inducing signals from the host [11]. The production of Pf filamentous phage by *Pseudomonas aeruginosa* biofilms results in the spontaneous assembly of a highly ordered liquid crystalline matrix that enhances biofilm function and thus bacterial fitness [12]. The excision of the CP4-57 prophage in a small fraction of cells at low temperature promotes the formation and survival of the biofilm in *Shewanella oneidensis*. This cryptic prophage excision serves as a regulatory mechanism that enables the host to survive at low temperature by regulating the activity of tmRNA and facilitating biofilm formation [13]. The *comK* gene in *Listeria monocytogenes* strain 10403S is interrupted by the φ10403S prophage. When prophage φ10403S is excised during phagocytosis, an intact *comK* gene is generated, and the repaired functional ComK protein activates the competence system (Com) system to induce phagosome escape [14]. Lysogeny endows the host with the ability to respond plastically to environmental changes, thus facilitating the host’s adaptation to extreme environments [15]. Furthermore, the integration of the phage genome confers superinfection exclusion, preventing infection of the same bacterial cell by closely related phages. Moreover, increasing evidence has shown that prophages are also reservoirs for various antiphage defence systems, protecting the host from lytic phage infection [16]. Additionally, prophages can mediate horizontal gene transfer (HGT) within hosts, thereby affecting the evolution, diversity, and biological characteristics of the bacterial community [17].

The phage–bacteria interaction in the ocean has been extensively explored in recent years [1, 18]. In particular, several studies have demonstrated that host availability and metabolic status are important factors that determine whether phage infection occurs via lytic dynamics or lysogeny, and lysogeny is in fact a successful strategy when bacteria are highly abundant and growing rapidly [19–21]. Reef-building corals harbour a high level of diverse commensal, mutualistic, or pathogenic microorganisms that inhabit the surface mucus layer, tissue, gastric cavity, and skeleton of corals [22, 23]. Thus, coral reefs represent a unique natural system for the study of lysis/lysogeny decisions in complex microbial communities. We recently found that the opportunistic coral pathogen *Vibrio coralliilyticus* outcompetes nonpathogenic commensal bacteria by using LodAB, which generates hydrogen peroxide. Specifically, this process triggers prophage induction of P2-like prophages in nonpathogenic *Vibrio* and other commensal strains [3]. The lysogenic-to-lytic switch of most other coral-associated bacteria has not been explored.

The *Halomonas* genus is a diverse group of halophilic bacteria renowned for their adaptability to high-salt environments, and they are widely distributed across various marine habitats [24, 25]. To cope with harsh conditions, *Halomonas* spp. utilize various mechanisms to enhance their survival primary through the production of bioactive components, which hold promise for applications in bioremediation, biopolymer production, and enzyme synthesis [26]. *Halomonas* spp. have been identified as prominent members or particularly enriched in some healthy reef-building corals such as in *Porites* spp. and *Acropora* spp. from the Mexican Caribbean and the Indo-Pacific regions [27–32]. *Halomonas meridiana* strains were isolated from surface mucus of *Acropora palmata* in the Caribbean area, and it can inhibit pathogens through production of antimicrobial compounds [33]. We previously found that *Halomonas* spp. were dominant among culturable bacteria isolated from the healthy reef-building coral *Galaxea fascicularis* in the South China Sea [34]. By studying a representative strain *H. meridiana* SCSIO 43005 (hereafter referred to as Hm43005) isolated from the gastric cavity of the *G. fascicularis*, we found that urea utilization is a common feature of *Halomonas* species [34]. Despite numerous analyses suggesting that environmental factors and stressors can result in a functional switch of the coral microbiota from a commensal to a pathogenic state, the factors determining the fate of coral-associated *Halomona*s spp. and the interplay between *Halomonas* and their prophages remain poorly understood.

In the present study, we characterized three prophages in Hm43005 and found that two prophages could be induced by the DNA-damaging agent MMC, leading to host death. Furthermore, Phm3 could amplify and package adjacent host DNA and attach to outer membrane vesicles (OMVs). Further analysis of the Tara Ocean dataset revealed that Phm3 represents a new group of temperate phages that are widely distributed and transcriptionally active in the ocean. Our findings have broadened the understanding of the impact of temperate phage-mediated lateral transduction events in marine ecosystems.

## Methods and materials

### Bacterial strains and growth conditions

The Hm43005, *E. coli* strains, and the plasmids used in this study are listed in Table S1. All the experiments with *E. coli* and Hm43005 were conducted at 37°C in Luria–Bertani (LB) medium and at 30°C in Marine Broth 2216E medium (BD Difco), respectively, unless otherwise indicated. DAP (2,6-diamino-pimelic acid) was added at a final concentration of 0.3 mM during the culture of *E. coli* WM3064. Chloramphenicol (30 μg·ml^−1^) was used for maintaining the pHGECm-based and pMBL*cas9*-based plasmids, ampicillin (100 μg·ml^−1^) was used to maintain the pUT18c-based and pBAD-based plasmids, and kanamycin (50 μg·ml^−1^) was used for maintaining the pK18*mobsacB*-based, pKT25-based, and pET28b-based plasmids in *E. coli* IPTG (isopropyl-β-D-thiogalactopyranoside) (1 mM) was used as an inducer.

### Construction of plasmids and mutant strains

The primers used in this study are listed in Table S2. The mutant strains of Hm43005 Δ*int*, Hm43005 Δ*rep*, Hm43005 *mcp*::*gfp*, Hm43005 *mcp*::*gfp* Δ*endolysin*, Hm43005 *mcp*::*gfp* Δ*holin*, Hm43005 *mcp*::*gfp* Δ*orf43*, and Hm43005 *mcp*::*gfp orf42* amber mutation were constructed using the suicide plasmid pK18*mobsacB* as reported previously [34]. The prophage deletion mutant strains ΔPhm1, ΔPhm3, and the double mutant strain ΔPhm1ΔPhm3 were constructed using the pMBL*cas9* plasmid as described previously [35]. The pHGECm vector was used to express lysis-related genes in the *E. coli* ER2738 strain. The pK18*mobsacB*-based and pMBL*cas9*-based plasmids were subsequently transferred into the target Hm43005 host from *E. coli* WM3064 by conjugation assays using a previously described method [34]. For localization, the *gfp* gene without a start codon was added to the N-terminus of the *orf42* gene, and the *mCherry* gene excluding the termination codon was added to the C-terminus of the *orf43* gene and subsequently cloned and inserted into pBAD and pHGECm. The constructed plasmids were all sequenced to ensure that no mutations were introduced during cloning.

### Phage purification

Phage particles were purified using the polyethylene glycol (PEG)–mediated precipitation method as previously described [3]. Briefly, Hm43005 and Hm43005 mutant cells were grown in Marine Broth 2216E medium to an OD_600_ of 1, MMC was added at 0.2 μg·ml^−1^, and the mixture was incubated for 24 h. The culture supernatant of the cells was collected by centrifugation (12 000 × g, for 45 min). The supernatant was supplemented with NaCl at a final concentration of 1 M and then incubated at 4 °C for 1 h. Subsequently, the supernatant was filtered through a 0.22-μm filter (Merck Millipore, Darmstadt, Germany) to remove bacterial cells but allow the passing of phages and then treated with DNase I (1 μg·ml^−1^) and RNase I (1 μg·ml^−1^) at 25 °C for 1 h. The filtrate was subsequently supplemented with PEG 8000 at a final concentration of 10% (m/v) and incubated overnight at 4 °C. The phage particles were pelleted by centrifugation at 12 000 × g for 45 min at 4°C, and the phage pellets were then resuspended in 1 ml of SM buffer (100-mM NaCl, 8-mM MgSO4, 50-mM Tris–HCl). Subsequently, the purified phage particles were negatively stained with 2% phosphotungstic acid (pH 6.8) and observed using an FEI G2 F20 TWIN electron microscope at 120 kV. A High Pure Viral Kit (Roche, Product No: 11858882001) was used to extract the Phm3 phage genome. The phage genomic DNA was sequenced with the sequencing platform (Illumina) and assembled by Shovill v.1.1.0 with default parameters (T. Seeman, https://github.com/tseemann/shovill). The Prophage Tracer was used to detect the *attP* site in the sequencing reads [36]. The phage genome termini were analysed by PhageTerm [37].

### Quantification of prophage excision and phage copy number

Hm43005 cells were grown in 2216E medium to an OD_600_ of 0.6, MMC was added at 0.2 μg·ml^−1^, and the mixture was incubated for 4 h. The cells were collected, and total DNA was extracted using a TIANamp Bacteria DNA Kit (Tiangen, Beijing, China). The frequency of prophage excision and the extrachromosomal phage copy number under different conditions were quantified by quantitative PCR (qPCR). The number of chromosomes that contained unoccupied *attB* was quantified using the primer set *attB*-qF/−qR, and the extrachromosomal circular form of the phage was quantified using the primer set *attP*-qF/−qR or *mcp*-qF/−qR. The total number of chromosomes was quantified using the single-copy reference gene *gyrB* with the primer set *gyrB*-qF/−qR.

### BACTH assay

Both pUT18C- and pKT25-based recombinant plasmids were cotransformed into *E. coli* BTH101 (cya-99) cells. After overnight culture, 10 μl of the cultures were added to MacConkey agar plates supplemented with 1-mM IPTG and 40 μg·ml^−1^ 5-Bromo-4-chloro-3-indolyl-β-D-galactopyranoside (X-gal). The plates were incubated for 24 h at 30°C.

### Phylogenetic analysis

For phylogenetic analysis of the Phm3 genome, 21 virus genomes that were relatively complete among the 50 best hits were collected through BLASTP searches of the National Center for Biotechnology Information (NCBI) virus database using the Phm3 major capsid protein (MCP) as the query. The genomes of these viruses and Phm3 were further subjected to genome-based phylogenetic analysis using the VICTOR [38] online server2 with default parameters. For Phm3 MCP phylogenetic analysis, phylogenetic trees were constructed from similar sequences using Mega 11 with the neighbour-joining method [39]. All the parameters were set to their defaults except for the bootstrap value, which was set to 1000; the p-distance model was used to calculate the distance; and the gap/missing data treatment cut-off was 50%.

### Observation of cell lysis and phage release by time-lapse fluorescence microscopy

Hm43005 *mcp*::*gfp* cells and cells with lytic module mutations were grown in 2216E medium to an OD_600_ of 0.6, and 0.2 μg·ml^−1^ MMC was added. Subsequently, the cells (1 μl) were immediately spotted on a microscope slide that was covered with 1% (w/v) agar slices and imaged using a fluorescence microscope (Carl Zeiss, Jena, Germany).

### Observation of outer membrane vesicles via fluorescence microscopy

Hm43005 *mcp*::*gfp* cells and cells with lytic module mutations were grown in 2216E medium to an OD_600_ of 0.6, 0.2 μg·ml^−1^ MMC was added, and the mixture was incubated for 4 h. One hundred microliters of the supernatant were stained with 800 nM FM4-64 membrane dye. Following incubation for 30 min at room temperature, the cells (1 μl) were spotted immediately on a microscope slide. A fluorescence microscope (Carl Zeiss, Jena, Germany) was used to detect the fluorescence signal of green fluorescent protein (GFP) or FM4-64 in the cells.

### RNA extraction and transcriptome sequencing

Hm43005 cells were grown overnight in 2216E medium with shaking at 26°C and 32°C. The cells were then collected, and total RNA was extracted using an RNAprep Pure Cell/Bacteria Kit (Tiangen, Beijing, China). Transcriptome sequencing was performed on a HiSeq 2500 platform (Illumina), and paired-end reads were generated.

### Statistical analysis

The data are presented as the means ± standard errors of the means (SEMs). All the statistical analyses were performed with GraphPad Prism 8 statistical software. Differences between two groups were evaluated using Student’s *t*-test. When three or more groups were compared in the experiment, multiple comparison was used if one-way analysis of variance showed statistical significance (*P* values < .05). *P* values < .05 obtained from multiple comparison and Student’s *t*-test were considered to indicate statistical significance.

## Results

### Genomic features of prophages in Hm43005

**Figure 1:**
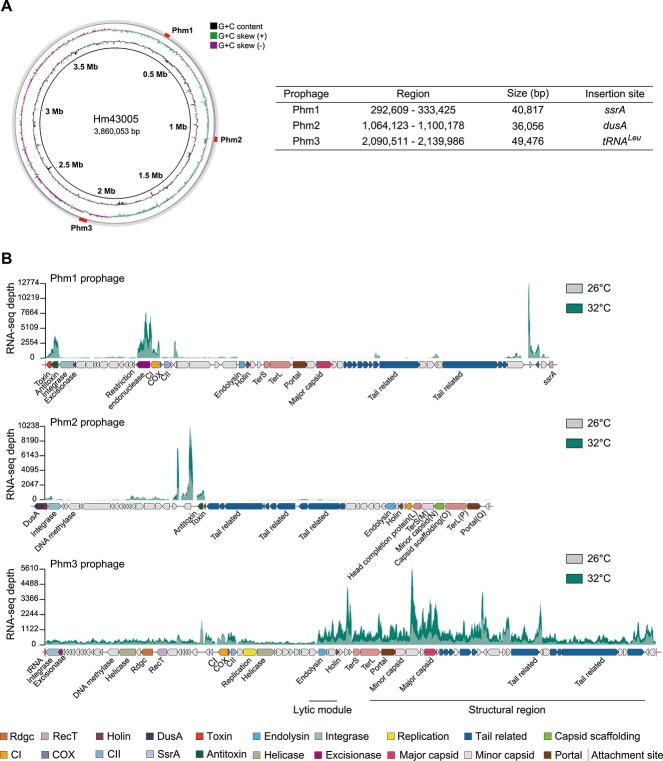
Phm3 is an active prophage in Hm43005. (A) Size and position of the three prophages in the genome of Hm43005. (B) Annotations of the Phm1, Phm2, and Phm3 prophages and expression levels of their encoding genes at 26°C and 32°C determined by a strand-specific transcriptome. The left and right attachment sites within the host chromosome are indicated by vertical bars at the ends of each prophages. The arrows represent the relative position and transcriptional direction of the ORFs. The predicted gene functions are indicated by the colour key at the bottom.

We first employed the Prophage Tracer, a tool for recognizing active prophages in prokaryotic genomes, to predict prophages in the genome of Hm43005 (Fig. 1A). Three putative prophages, Phm1, Phm2, and Phm3, were identified based on the presence of discriminative reads that contained bacterial (*attB*) and phage (*attP*) attachment sites representing prophage excision signals. Further phage genome annotation revealed capsid, tailed, and lysis-related genes in all three prophages (Fig. 1B**;**Tables S3), suggesting that these prophages should retain their capacity to assemble into phage particles and lyse the host upon induction.

Prophage Phm1 has a 40 817-bp genome with 54 predicted genes inserted in the tmRNA (*ssrA*) gene that serve as a hotspot for prophage integration into a variety of Gram-negative bacteria (Fig. 1B). The MCP of Phm1 shares 99.3% sequence identity with that of the temperate phage vB_HmeY_H4907, which infects the *H. meridiana* H4907 strain isolated from the surface sediment of the Mariana Trench at a depth of 8900 m (Table S3) [40]. Prophage Phm2 possesses a 36 056-bp genome containing 48 predicted genes; this genome is integrated at the 5′-end of the *dusA* gene that encodes tRNA-dihydrouridine synthase. The integrase of Phm2 is a *dusA*-associated integrase, a specific type of tyrosine recombinase shared by various mobile genetic elements integrated within the *dusA* gene [41]. Phm2 contains a conserved diagnostic signature region of the P2-family structural region, L-M-N-O-P-Q (from CTT34_*05375* to CTT34_*05400*) (Fig. 1B**;**Table S3) [42], and also carries lysis-related genes that encode endolysin, holin, and Rz-like spanin. Prophage Phm3 has a 49 500-bp genome with 77 predicted genes that is integrated within the 3′-end of the *tRNA^Leu^* gene. Most of the Phm3 genes lack annotations and encode hypothetical proteins without a valid Pfam domain (Fig. 1B; Table S3).

To probe the activity of these prophages, we conducted a transcriptome analysis. Because heat stress is a major factor that causes coral bleaching, we analysed the transcription of prophage genes in Hm43005. In our previous study, we have demonstrated that when the reef-building coral *G. fascicularis* was infected with coral pathogen *V. coralliilyticus*, it caused severe tissue loss at 32°C but not at 26°C [3]. Thus, these two temperatures were selected for the transcriptome analysis. The phage structural region and lysis-related genes of Phm1 and Phm2 were silenced, and only a few phage repressor genes and toxin–antitoxin genes were expressed. In contrast, the phage structural region and lysis-related genes of Phm3 were expressed at 26°C, and the expression of these genes increased ~3-fold when the temperature was increased to 32°C (Fig. 1B**;**Tables S4). These results demonstrated that Phm3 is the most active prophage among the three prophages in Hm43005.

### Phm3 induction potentially increases host DNA mobility via lateral transduction

**Figure 2:**
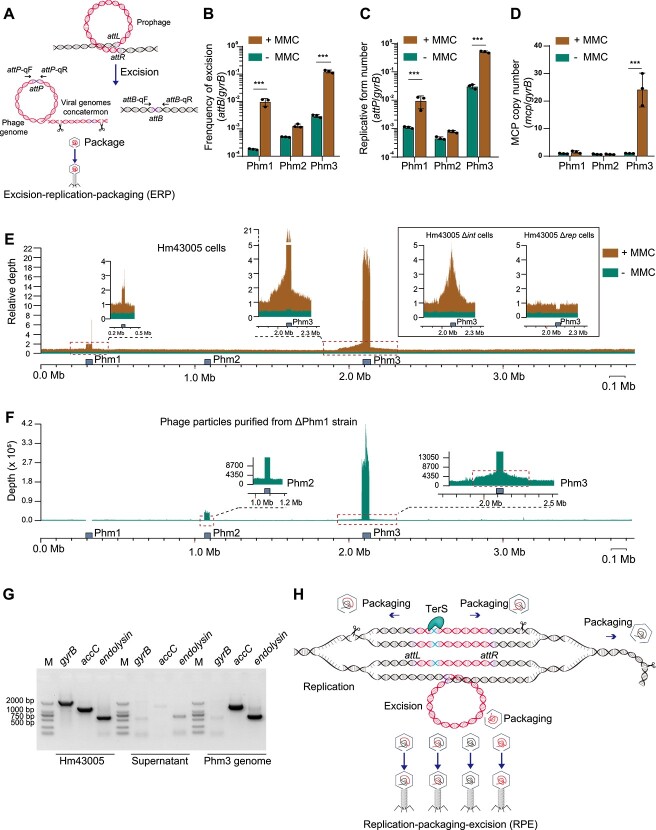
Phm3 is an SOS-inducible temperate phage that can package adjacent host DNA. (A) Schematic diagram illustrating the prophage genome excision of Phm1 via the canonical ERP pathway. The sequences of primers used for the amplification of *attB* and *attP* are also shown. The excision form (B) and replicative form (C, D) of the three prophages were quantified in the presence or absence of MMC. Three independent cultures were used, and the data are shown as the mean ± SEM (*n* = 3). Significant changes with ^***^P < .001 are marked with three asterisks. (E) Whole-genome sequencing of Hm43005 cells treated with and without MMC. The average depth was calculated by the R/Bioconductor package karyoploteR. Whole-genome sequencing of Hm43005 Δ*int* and Hm3005 Δ*rep* cells with and without MMC. The average depth was calculated by the R/Bioconductor package karyoploteR. (F) Whole-genome sequencing of phage particles purified from the Hm43005 ΔPhm1 strain after treatment with MMC. (G) PCR was used to detect the *accC* gene located 10 kb upstream of prophage Phm3, the endolysin gene located at prophage Phm3, and the *gyrB* gene located 2 Mb upstream of prophage Phm3. (H) Schematic diagram illustrating that Phm3 functions via the noncanonical RPE [27] pathway. The Phm3 prophage genome replicates *in situ*, and the adjacent bacterial DNA is amplified before its excision. The DNA packaging machinery then proceeds to package additional headfuls of bacterial chromosomes spanning several hundred kilobases.

To monitor the lysogeny–lysis conversion of the prophage, the excision rate of the three prophages or the replication of the circularized prophage circle were quantified via quantitative PCR (qPCR) by comparing the number of *attB* sites (present only after prophage genome excision) or the number of *attP* sites (present only in circular phages) to the number in the single-copy chromosomal gene *gyrB*, respectively. In parallel, prophage replication was also quantified by comparing the phage MCP gene and the *gyrB* gene (Fig. 2A).

Under normal growth conditions, Phm1 was maintained in a lysogenic state, with an excision rate of ~1 out of 10 000 cells (Fig. 2B). After MMC treatment (MMC, 0.2 μg·ml^−1^), which triggers the host SOS response, the excision rate of Phm1 increased 55 ± 15-fold (Fig. 2B). The *attP*/*gyrB* ratio of Phm1 also increased after MMC treatment (Fig. 2C). Previous studies demonstrated that excision of the P4-family prophage in *Shewanella* sp. and in *E. coli* disrupts *ssrA* function by causing loss of G:U wobble base pairing at its 3′-end [13, 43]. However, genome excision of Phm1 did not affect the wobble base pairing of SsrA [36]. The frequency of Phm2 excision was similar to that of Phm1 excision. However, Phm2 was a noninducible prophage because MMC did not induce excision of Phm2 (Fig. 2B and C). This result is consistent with our previous finding of a P2-family prophage in deep-sea-derived *Shewanella* sp. W3-18-1 [44].

Among these prophages, Phm3 was the most active prophage. The qPCR results revealed that the estimated frequency of Phm3 excision was 3 out of 1000 cells under normal growth conditions (Fig. 2B). After treatment with MMC, the excision rate of Phm3 increased 43 ± 0.7-fold, resulting in Phm3 excision in nearly 50% of the cells. For Phm3, the number of circularized prophages per cell (*attP*/*gyrB*) was also significantly higher after treatment with MMC (Fig. 2C). Phm3 phage replication (*mcp*/*gyrB*) was also induced by MMC treatment (Fig. 2D). Resequencing of the genome of Hm43005 host cells under normal growth conditions showed that the Phm3 prophage region had a slightly greater sequence depth than the remaining genomic region. After treatment with MMC, the Phm1 prophage region presented an average sequence depth that was ~3-fold greater than that of the remaining genomic region, whereas the Phm3 prophage region exhibited an average sequence depth that was ~20-fold greater than that of the remaining genomic region (Fig. 2E). Collectively, these results showed that Phm3 is an active and SOS-inducible prophage in Hm43005.

Unexpectedly, the sequencing depths of the flanking regions of prophage Phm3 were unusually higher than those of other host genomic regions of Hm43005 cells treated with MMC (Fig. 2E). The host genome flanking the Phm3 prophage showed considerable amplification and spanned up to ~200 kbp upstream and downstream of the prophage, which included genes involved in various metabolic pathways, including carbohydrate metabolism, type IV pilus assembly, and glycerol transport (Table S5). The host genome was amplified up to 3-fold, and the sequencing depth gradually decreased far away from the prophage region (Fig. 2E). To further verify this finding, we sequenced the Phm2 and Phm3 phage particles collected from the ΔPhm1 mutant strain. Indeed, unlike Phm2, which contains only its genome, the Phm3 phage genome appeared to contain the host genome flanking the prophage attachment sites (Fig. 2F), suggesting that Phm3 phage particles could package the host genome DNA. The PCR test of phage DNA specifically detected the endolysin gene (CTT34_*10225*) located at prophage Phm3, the *accC* gene (CTT34_*09970*) that located 10 kb upstream of prophage Phm3, but not the *gyrB* gene (CTT34–*00020*), located 2 Mb upstream of prophage Phm3, which further confirmed the packaging of flanking host genome in Phm3 phage particles (Fig. 2G). This aberrant prophage excision of Phm3 is similar to the lytic cycle of staphylococcal prophages, which can transfer prophages adjacent to host genes through lateral transduction [45, 46]. The staphylococcal prophages exhibit delayed excision that occurs late in their lytic cycle, and this excision is followed by a replication–packaging–excision (RPE) pathway. This pathway results in the amplification and packaging of neighbouring host DNA and differs from the classical excision–replication–packaging (ERP) pathway (Fig. 2A). Furthermore, the copy number of the *mcp* gene was markedly higher than the number of the replicative form of Phm3 prophage (Fig. 2D), suggesting that phage genome circularization may only partially occur during the induction of the Phm3 prophage. This finding is consistent with the fact that the circularized phage genome density was low at the early stage of the RPE pathway [46]. To investigate whether Phm3 release followed the RPE pathway, we deleted the integrase gene (*int*) that is required for prophage excision and the replication gene (*rep*). Deep sequencing was performed for the two deletion mutants with and without MMC (Fig. 2E). Upon MMC treatment, deleting *int* maintained the ability to amplify the prophage region and the flanking host DNA region, whereas deleting *rep* abolished the amplification, indicating that the prophage-encoded replication protein can amplify host DNA *in situ*. These results collectively indicate that Phm3 is an SOS-inducible phage with an RPE induction cycle that can amplify and package adjacent host DNA and can lead to HGT via lateral transduction (Fig. 2H).

### The Phm3 phage represents a new group of temperate phages in the ocean

**Figure 3:**
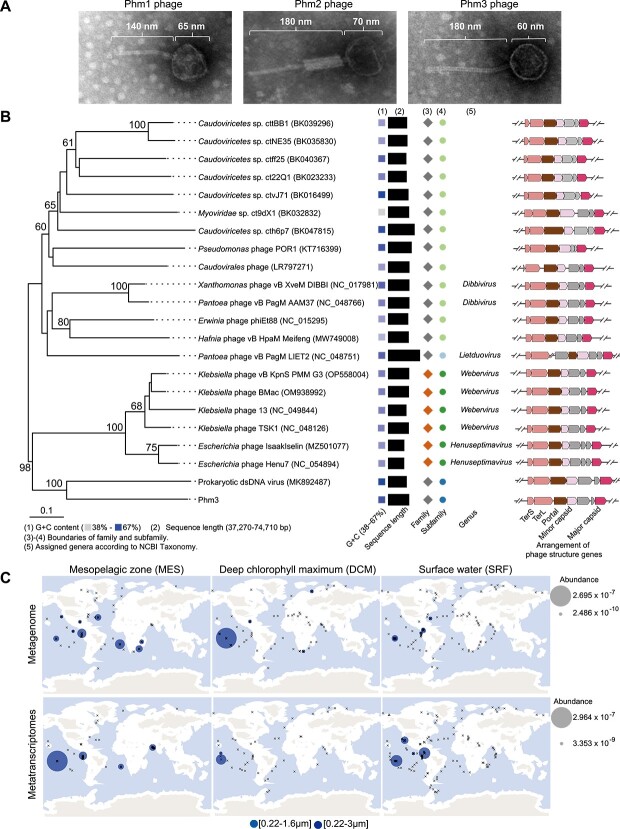
Phylogenetic analysis of Phm3 based on the genome sequences of phages. (A) Transmission electron micrograph of the phages Phm1, Phm2, and Phm3. (B) Phylogenetic analysis of the genome sequence of Phm3 using VICTOR (formula D0). The family and subfamily boundaries were derived from the VICTOR evaluation results. The squares represent families, the circles represent subfamilies, and the different colours indicate different families/subfamilies. Genus names were derived from NCBI. Scale bar, 0.1 substitutions per amino acid position. (C) Distribution of Phm3-like temperate phage MCP-encoding genes in the Tara Ocean dataset. In the Ocean Gene Atlas web server, the amino acid sequence of Phm3 was used as the query for searching the OM-RGC_v2_METAg and OM-RGC_v2_metaT datasets with thresholds of 1e-60 and 1e-48, respectively.

Transmission electron microscopy (TEM) of the supernatant from Hm43005 cells treated with MMC revealed phage particles with different morphologies, indicating that more than one prophage can assemble into virion particles upon induction (Fig. S1). To further confirm the morphology of the individual phages, we constructed two single-prophage-deletion mutant strains (ΔPhm1 and ΔPhm3) and one double mutant ΔPhm1ΔPhm3 strain (Fig. S2), and the phages were isolated by PEG precipitation. Because Phm2 was not inducible by MMC treatment, the ΔPhm1 and ΔPhm3 strains treated with MMC were used to purify Phm3 and Phm1, respectively. The mutant strain ΔPhm1ΔPhm3 was used for the isolation of Phm2. The integrated and replicative forms of the respective prophage genomes were precisely removed from the Hm43005 genome using the CRISPR/Cas9 system that we developed [35]. TEM revealed that Phm1 shares morphological similarity with the λ phage and has an isometric icosahedral head (~65 nm in diameter) and a long, flexible tail (~140 nm in length and 10 nm in width). Phm2 exhibits morphological similarity to the P2 family of phages, belongs to myovirus and has a rigid tail (~180 nm in length and 20 nm in width). Phm3 also belongs to the siphovirus family and possesses an isometric icosahedral head (~60 nm in diameter) and a longer flexible tail than Phm1 (~180 nm in length and 10 nm in width) (Fig. 3A).

Similar to many previously reported phages of the *Siphoviridae* family, Phm3 also has many genes with unknown functions [47, 48]. Among the 77 open reading frames (ORFs) predicted, only 37 were annotated with a designated function (Fig. 1B; Table S3). The predominant start codon was AUG (68 ORFs), but there were also incidences of alternative start codons, i.e. UUG (ORF74) and GUG (ORF19, 20, 37, 38, 42, 53, 62, 76). No tRNA or rRNA gene was found. These predicted proteins were compared with those in the Conserved Domains Database (CDD) and can be roughly classified into four major categories: structural, lytic/lysogenic, regulatory, and other genes.

The Phm3 phage structural protein-coding genes cluster in a module and are preceded by a terminase gene. ORF50 is predicted to be the portal protein, which plays a distinctive role in the assembly of double-stranded DNA (dsDNA) bacteriophages. Along with its role in regulating procapsid assembly, various bacteriophages function as DNA sensors, facilitating both genome packaging and release [49]. ORF54 is predicted to encode the MCP, and ORF51 is predicted to encode the MCP. The phage tail is a very complex structure that plays an important role in host recognition, attachment, and cell membrane penetration [50]. A total of 9 out of 37 proteins with known activities were identified as tail-associated proteins. ORF58 encodes the major tail protein (MTP), which is the main component of the long noncontractile phage tail [51], and ORF60 encodes the tail tube protein (TTP), which plays an important role in the injection of DNA [52]. In addition, successful assembly of the tail requires the presence of a tail assembly chaperone (TAC; encoded by ORF61) [53]. The tape measure protein (TMP; encoded by ORF67) determines the length of the tail tube [54]. ORF69 encodes the distal tail protein, which forms a hexameric ring during tail assembly, and this structure acts as a hub for distal tail adsorption and tail tube formation [55]. ORF70 encodes a baseplate, which is a key component of the tail that mediates host binding and genome injection [56]. ORF71 encodes a minor tail protein that also plays an important role in the tail assembly of bacteriophages [57]. ORF72 and ORF75 encode two tail fibre proteins that can be assembled with the help of chaperones to correct phage mature fibres, which are responsible for specific, albeit reversible, primary attachment to host cells [58].

To determine the taxonomic status of Phm3, 21 virus genomes, which were relatively complete among the 50 best hits, were collected through BLASTP searches of the NCBI virus database using the Phm3 MCP as the query. The genomes of these viruses and Phm3 were further subjected to genome-based phylogenetic analysis using VICTOR [38]. According to the VICTOR-constructed phylogenetic relationship, Phm3 was found to be most closely related to a metagenomic assembled prokaryotic dsDNA virus (MK892487.1) obtained from the virome during the Tara Oceans and Malaspina research expeditions (Fig. 3B**;**Table S6) [59]. Phm3 and the uncultured prokaryotic dsDNA virus formed a subfamily level cluster, and the genera of these viruses were not assigned according to the NCBI taxonomy. Similarly, a phylogenetic tree constructed using the MCP amino acid sequences showed that Phm3 was clustered with prophages belonging to the *Halomonas* genus, indicating that Phm3-like temperate phages infect various *Halomonas* species (Fig. S3). Phm3-like prophages were also found in *Kushneria*, *Bordetella*, and *Cupriavidus* species (Fig. S3**;**Table S7). Further comparative genomic analysis revealed that the Phm3-like temperate phage genome contains a conserved region encoding the portal protein, minor capsid protein, capsid fibre protein, and MCP (Fig. S4A and B). Furthermore, to explore whether other Phm3-like temperate phages follow the RPE pathway, we analysed the replication-related proteins. Initiator-helicase loader (IL-type) and initiator-helicase (IH-type) are typical replication modules [60]. Many temperate phages (including the prokaryotic dsDNA virus) possess IL-type or IH-type replication modules with replication initiation proteins similar to the λ replication protein O that is capable of theta (θ)-type DNA replication (Fig. S4C) [60]. We further extended this analysis to other reported prophages capable of amplifying adjacent host DNA [45, 46] and our previously identified MMC-inducible prophages in other coral-associated bacteria. The reported prophages with RPE pathways (e.g. *Staphylococcus* phage 80α, φ11, φ52A, *Salmonella* phage P22, and *Enterobacteria* phage ES18) all possess IL-type or IH-type replication modules with replication proteins similar to λ replication protein O or a recently identified replication initiator, featuring a conserved N-terminal domain of HTH superfamily (Fig. S4D) [61]. To further explore the importance of the replication module in mediating lateral transduction, we selected three prophages (Phm1, Pzm1, and Pea1) in coral-associated bacteria that were unable to amplify adjacent host DNA [36]. Our analysis revealed these prophages possess replication proteins with different domain architectures (Fig. S4E). These analyses suggest that the replication modules of temperate phages might be critical for phage-mediated lateral transduction events.

MCP was then used to explore the distribution of Phm3-like temperate phages in other marine environments. As revealed through a search of the Tara Ocean datasets*,* Phm3 MCP homologues (>75% amino acid identity) were found mainly in the deep chlorophyll maximum (DCM) and mesopelagic zone water samples (Fig. 3C). Furthermore, these MCP-encoding genes were transcribed in these samples and were derived from *Halomonas*, suggesting that Phm3-like temperate phages harbouring *Halomonas* strains may be widely distributed (Fig. 3C). Taken together, these findings suggest that Phm3 may represent a new group of temperate phages that are widely distributed in various marine environments.

### Host cell lysis by Phm3 is accompanied by outer membrane vesicle production

**Figure 4:**
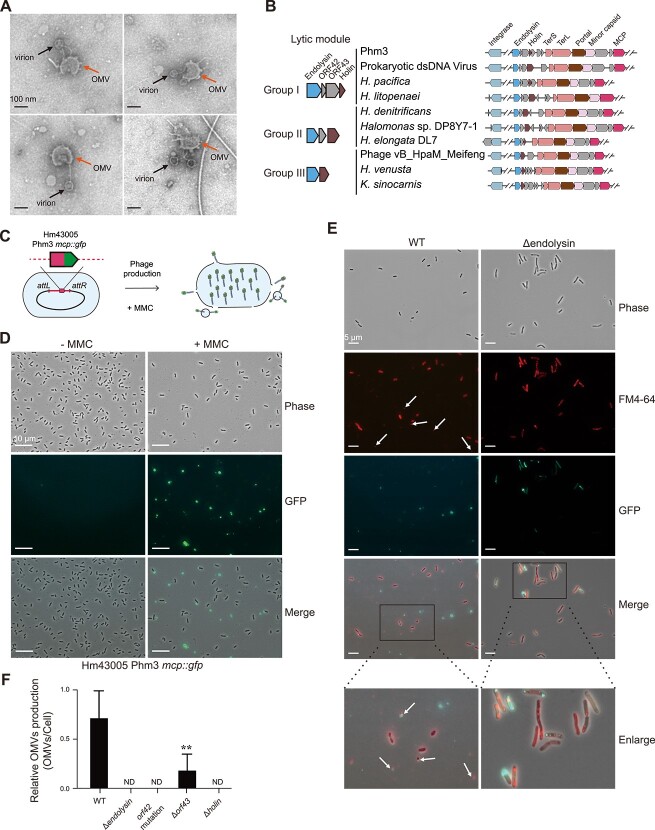
The release of Phm3 is accompanied by the formation of OMVs. (A) Flexible tails of Phm3 phages were inserted into OMVs. (B) Analysis of the lytic module in Phm3-like temperate phages. The genes are presented by block arrows and are coloured according to their function. (C) Schematic of the reporter system Hm43005 *mcp*::*gfp* for visualization of Phm3 induction. (D) The reporter strain Hm43005 Phm3 *mcp*::*gfp* was utilized to visualize the induction of Phm3 in host cells. The induction of Phm3 was visualized by intracellular MCP-GFP expression. (E) The adsorption of Phm3 phages to OMVs was visualized via fluorescence microscopy. The production of the Phm3 phage induces the production of more OMVs from wild-type Hm43005 than from the mutated lysis-related genes. The membranes were stained with 800 nM FM4-64 membrane dye. (F) Quantification of OMV production in the wild-type and mutant Hm43005 strains. ImageJ software was used. The data are shown as the mean ± SEM. Each group was compared using an unpaired *t*-test with 95% confidence intervals (*n* = 3), and significant changes with *P* < .01 are marked with two asterisks.

The TEM examination of phage particles in the culture supernatant revealed a substantial number of OMVs (Fig. S1). We also observed that flexible tails of some virions of Phm3 were inserted into OMVs (Fig. 4A), and some OMVs with inserted phage tails exhibited an irregular morphology. We reasoned that the release of Phm3 may affect the OMV production of Hm43005.

Phage release–related host cell lysis is involved in the subversion of the inner membrane, peptidoglycan, and the outer membrane. Comparative analysis of Phm3-like phages revealed that these phages have a relatively conserved structural module but various lytic modules, and these lytic modules can be categorized into three main types (Fig. 4B). Group III members consist of only endolysin and holin, whereas Group I has two additional proteins, and Group II has one additional protein. For the release of progeny virions, *Caudovirales* use “multigene lysis” systems that involve at least two proteins, holin and endolysin [62]. The lytic module of Phm3 belongs to Group I and contains four proteins, ORF41-ORF44 (Figs 4B and S5A). ORF41 encodes an endolysin protein that harbours a conserved EGGY motif near the N-terminus, and the glutamic acid within this motif is essential for catalytic activity [63]. Additionally, endolysins have no signal sequence (Fig. S5A and B). In addition, endolysin was expressed and purified (Fig. S5C). ORF44 encodes holin, which can interact with the host cell membrane to allow endolysin to penetrate the bacterial cell wall [64]. Analysis of the primary sequence of holin suggested a simple membrane topology with two membrane-spanning helical domains and a highly charged, hydrophilic C-terminus (Fig. S5A). ORF42 and ORF43 are located between the *endolysin* and *holin* genes; one of these genes encodes a hypothetical protein that contains membrane-spanning helical domains in the C-terminus, and the other encodes a protein containing a signal sequence in the N-terminus and a DUF3761 domain in the C-terminus (Figs 4B and S5A).

To investigate the relationship between phage lysis and OMV production, the fluorescently labelled strain Hm43005 Phm3 *mcp*::*gfp* in which the *mcp* gene was fused in frame with the *gfp* gene was constructed (Fig. 4C). Because MCP is needed for the assembly of phage particles, the expression of fused MCP::GFP can be used as an indicator of Phm3 phage production. As expected, non-MMC-treated cells appeared dim under a fluorescence microscope, suggesting that Phm3 was mostly maintained in a lysogenic state under normal growth conditions. After treatment with 0.2 μg·ml^−1^ MMC for 4 h, a high proportion of cells produced Phm3 phage particles (Fig. 4D), confirming that MMC treatment can be used to study the process of Phm3 induction. In parallel, for the GFP-labelled strain, four mutants in which each of the four lysis-related genes were individually deleted or disrupted were constructed, yielding the Δ*holin*, Δ*endolysin*, Δ*orf43*, and *orf42* amber mutation strains. MMC-induced Hm43005 Phm3 *mcp*::*gfp* and lysis-related mutant cells were stained with the lipophilic fluorescent dye FM4-64 for 30 min. The presence of OMVs, which can be stained with FM4-64 to emit red fluorescence, was observed in the MMC-treated Hm43005 Phm3 *mcp*::*gfp* strain caused by phage efflux (Fig. 4E). Our data demonstrated significant inhibition of phage release in the mutant strains, and this inhibition was accompanied by a substantial decrease in OMV production (Fig. 4F). Although Phm1 was induced in the Hm43005 Phm3 *mcp*::*gfp* strain, mutation of any of the lysis-related genes of Phm3 was sufficient to reduce OMV production. Therefore, these results indicate that Phm3 phage-triggered cell lysis plays a major role in the production of OMVs.

### The lytic module of Phm3 mediates phage release and outer membrane vesicle production

**Figure 5:**
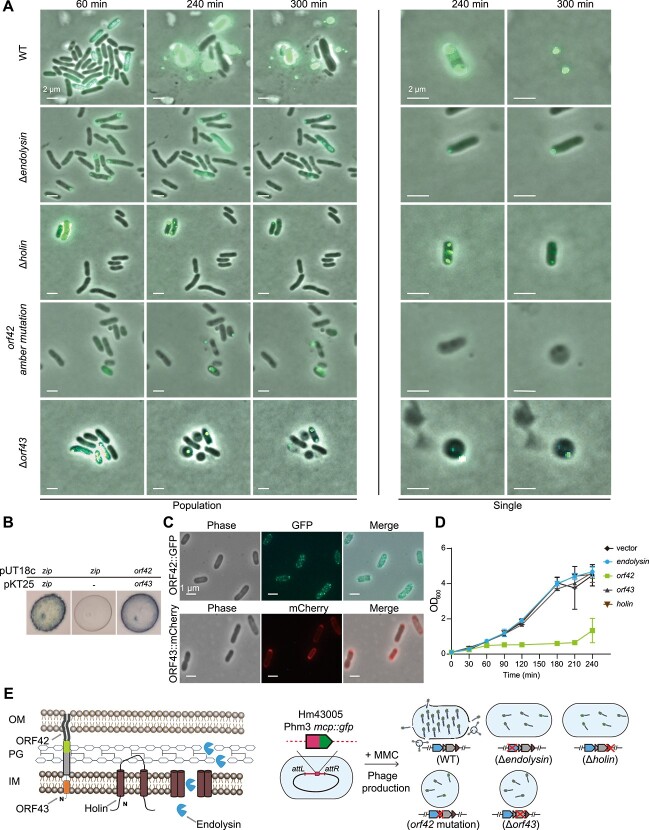
New lytic module composed of four genes controlling cell lysis and phage release. (A) Fluorescence microscopy observation of Phm3 induction. Overlay images of phase contrast and the GFP channel depicting the induction of Phm3 in Hm43005 *mcp*::*gfp* cells. Single-cell and cell-population analyses showed that phages can be released and cause host lysis after induction for 240 min in wild-type Hm43005. The deletion of *endolysin* and *holin* obstructed the release of the Phm3 phage. The amber mutation in *orf42* and the deletion of *orf43* have impacts on both Phm3 production and cell morphology. (B) The interaction between ORF42 and ORF43 was analysed by bacterial two-hybrid analysis. T18/T25 were included as a positive interaction. (C) Fluorescence microscopic localization of ORF42 and ORF43 in *E. coli* cells. (D) Effect of lysis-related gene overexpression on the growth of *E. coli*. (E) The predicted topologies of proteins encoded by lysis-related genes are shown on the cell membrane, and the reporter system Hm43005 *mcp*::*gfp* was generated for the visualization of Phm3 induction in the wild-type and lytic module-mutated strains.

The Δ*holin*, Δ*endolysin*, Δ*orf43*, and *orf42* amber mutation strains were subjected to time-lapse fluorescence microscopic examination using an agarose pad. Subsequently, we use the time-lapse microscopy to monitor a cell undergoing induction with Phm3 promoted cell rupture to produce MCP (Fig. 5A). After the addition of MMC, Hm43005 cells were lysed for 120–240 min, and the rod-shaped cells were able to proliferate and released phage particles upon host lysis (Fig. 5A). In contrast, in the absence of holin or endolysin, the induced Phm3 phage appeared to be stuck near the cell poles or in the middle, and the host cells remained rod-shaped and lost the ability to rupture (Fig. 5A). Similar to most *Caudovirales* phages, during release of the Phm3 phage, holins form pores in the cytoplasmic membrane, allowing the release of endolysins into the periplasmic space to hydrolyse peptidoglycan in the periplasmic space [63].

We also observed that when ORF42 or ORF43 was disrupted, the rod-shaped host cells still became bloated and rounded after 120 min when Phm3 was induced to form phage particles. However, the release of phages was apparently delayed because many of these rounded cells were unable to undergo cell lysis to release phage particles, a well-controlled cascade, as observed with the Hm43005 host cells. When Phm3 was induced and phage particles were assembled, the action of endolysin was triggered to disrupt the peptidoglycan layer, leading to the formation of spheroplasts without ORF42 or ORF43. These data collectively suggest that ORF42 and ORF43 facilitate disruption of the outer membrane to successfully complete the phage release process. Thus, the two proteins located between holin and endolysin act in a similar manner as spanins in temperate λ phage and may play a crucial role in disrupting the host outer membrane during phage release and host cell lysis.

The spanin complex completes host lysis by rupturing the outer membrane after the actions of holin and endolysin penetrate the inner membrane and break down the host peptidoglycan. To determine whether ORF42 and ORF43 function as spanins, we tested whether they could form a complex. A bacterial two-hybrid system based on complementation of the T18 and T25 fragments of adenylate cyclase [65] was used. We fused *orf42* and *orf43* to the T25 and T18 fragments, respectively, and coexpressed the gene fusions in *E. coli* BTH101. We observed a strong interaction between ORF42 and ORF43, indicating that they likely form a complex (Fig. 5B). To further determine the localization of the proteins, we produced ORF42 tagged with GFP at the N-terminus and ORF43 tagged with a red fluorescent protein (mCherry) at the C-terminus (Fig. 5C). The localization of GFP-fused membrane proteins at the cell surface, as previously observed with the characterized membrane protein OmpA [66], suggested that the protein ORF42 is anchored in the outer membrane and acts as an o-spanin. Additionally, the red fluorescence produced was evenly distributed across the cell membrane, consistent with the distribution of other inner membrane proteins [67], indicating that the ORF43 is anchored in the inner membrane and functions as an i-spanin. Because the lytic module plays a direct role in bacterial lysis, we also explored the toxic effect of these lysis-related genes by ectopically expressing them in *E. coli*. We found that overproduction of endolysin, holin, or i-spanin did not affect the growth of *E. coli*, but overproduction of o-spanin significantly inhibited the growth of *E. coli* (Fig. 5D). Overall, the lytic module composed of the endolysins *o*-spanin, i-spanin, and holin functions cooperatively to lyse host cells and release Phm3 (Fig. 5E).

## Discussion

The coral microbiome includes dinoflagellates, viruses, fungi, archaea, and bacteria, whose spatial and temporal shifts are closely related to coral acclimatization and adaptation [68]. Temperate phages have been found to exhibit a tendency towards lysogeny in high-density microbial-communities, including the coral microbiome [19–21]. Lysogens in the coral microbiome are also affected by environmental and host factors. However, whether these lysogens can be converted to lysates and how these processes affect the microbial community are not well known. Herein, we identified three prophages in a coral-associated Hm43005 strain. Two of these prophages could be induced in response to DNA damage, which can be caused by common factors in coral reef environments, such as ultraviolet (UV) light, oxybenzone (sunscreen), and microplastics [69, 70]. RNA-Seq analysis revealed that only several genes, such as repressor and toxin-antitoxin genes, were expressed in the prophages Phm1 and Phm2, suggesting that these two prophages were silenced. In contrast to Phm1 and Phm2, Phm3 exhibited spontaneous activation, and the expressions of Phm3 phage structural genes were increased at elevated temperature. Considering that environmental factors, especially elevated seawater temperature, lead to an increase in coral disease occurrences [71], we reasoned that the production of Phm3 phage contributes to changes in the coral microbiome, ultimately leading to dysbiosis within the commensal microbial community.

In this study, we have identified a new group of temperate phages within coral-associated *Halomonas* species capable of facilitating lateral transduction. Lateral transduction, a form of HGT mediated by prophages, was initially observed in human-associated lysogens such as *Staphylococcus* and *Salmonella* strains [45, 46, 72]. Furthermore, our investigation revealed that IL-type or IH-type replication modules may be play a crucial role in prophages capable of lateral transduction. These replication modules were also identified in other marine temperate phages infecting different host bacteria, especially in the TARA Ocean–derived prokaryotic dsDNA virus. Moreover, an additional characteristic of Phm3 is its ability to attach to OMVs. OMVs have been demonstrated to package and transport diverse cargos, including DNA and bioactive molecules, playing vital roles in HGT and nutrient acquisition [73]. This suggests a potential alternative mechanism for Phm3 infection through hitchhiking on OMVs. Thus, our findings extend the understanding of temperate phage-mediated lateral transduction events beyond human pathogens to encompass marine microbes, highlighting their broader impact in diverse ecosystems.

Prophage endolysin–triggered explosive cell lysis has been proposed as a new mechanism for membrane vesicle production [74]. Stresses, including ciprofloxacin and MMC, can induce and promote explosive cell lysis events [75]. In this study, we found that the release of Phm3 was accompanied by OMV production, which was not observed during Phm1 or Phm2 phage release. Phm3 possesses a new lytic module containing endolysin, o-spanin, i-spanin, and holin, and disruption of any of these proteins significantly reduced OMV production, suggesting that an intact lysis box is needed for OMV production during Phm3 release. A previous study showed that overexpression of the endolysin gene encoded within the R- and F-pyocin gene cluster could cause cell lysis [76], and endolysin is the major trigger of explosive cell lysis [73, 74]. Although endolysin and holin were also present in the Phm1 and Phm2 phage genomes, the release of the Phm1 and Phm2 phages did not increase OMV production. A previous study showed that phages infecting a specific host may have highly diverse phage endolysins and that horizontally transferred phage endolysins could evolve and adapt to new hosts [77]. Collectively, these findings suggest that only a specific lytic module or conditional (programmed) expression of genes within the lysis box can trigger OMV production. Thus, the newly characterized lytic module facilitates OMV production during Phm3 release, potentially broadening our understanding of the ecological impact of marine temperate phages.

## Supplementary Material

Supplementary_file_wrae085

Table_S3_wrae085

Table_S4_wrae085

Table_S5_wrae085

Table_S6_wrae085

Table_S7_wrae085

Dataset_1_wrae085

## Data Availability

Our sequences files of transcriptome are accessible from the National Center for Biotechnology Information (BioProject PRJNA416452) and all the other data supporting the conclusions of this study is available in the paper and supplemental materials.
