# Supplementary material for: Active prophages in coral-associated Halomonas capable of lateral transduction
Source: ISME J. 2024 May 13;18(1):wrae085. doi: 10.1093/ismejo/wrae085 (PMC11131426; doi:10.1093/ismejo/wrae085)
Supplement: Supplementary_file_wrae085 [file supplementary_file_wrae085.docx]

**Supplementary File**

**Active prophages in coral-associated *Halomonas* capable of lateral transduction**

Ziyao Liu^1,2^, Kaihao Tang^1,2,3*^, Yiqing Zhou^1^, Tianlang Liu^1,2^, Yunxue Guo^1,2,3^, Duoting Wu^1^, Xiaoxue Wang^1,2,3*^

^*^Correspondence should be addressed to: [xxwang@scsio.ac.cn](mailto:xxwang@scsio.ac.cn); [khtang@scsio.ac.cn](mailto:khtang@scsio.ac.cn)

**This PDF file includes:**

Figures S1 to S5

Tables S1 to S2

Legends for Tables S3 to S7

SI References

**Other supplementary materials for this manuscript include the following:**

Tables S3 to S7


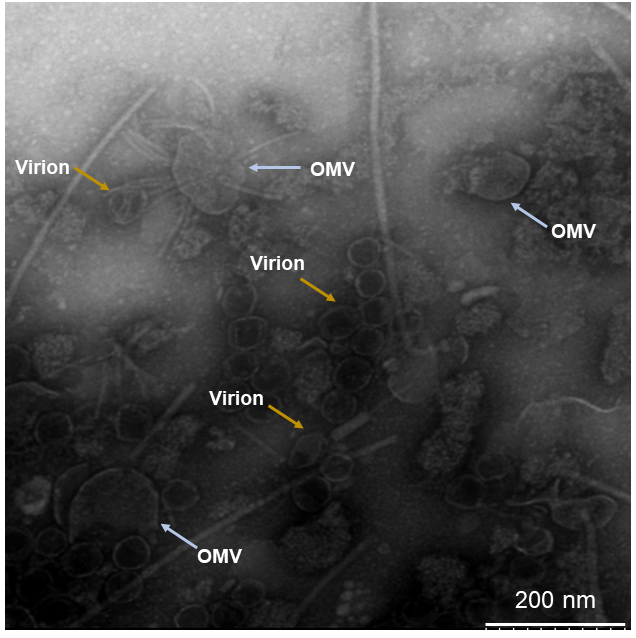


**Figure S1. TEM image of the Hm43005 supernatant in the presence of MMC.**

Virions and OMVs of different morphologies were observed in the supernatant. Different phage particles are indicated by arrows of different colors.


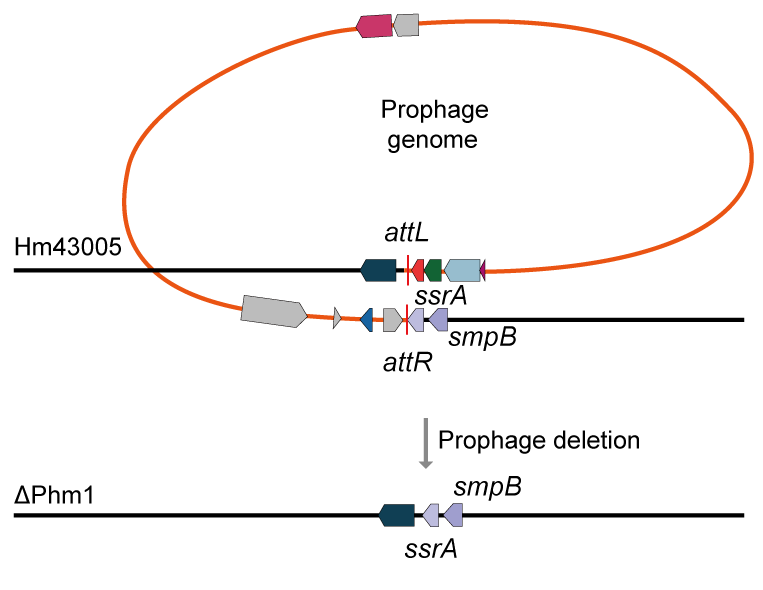


**Figure S2. Schematic representation of prophage Phm1 deletion in Hm43005.**

The CRISPR/Cas9 system [1] was used to remove the prophage Phm1 from the Hm43005 genome. The red curve represents the Phm1 prophage genome. The attachment sites *attL* and *attR* within the host chromosome are indicated by red vertical bars. The arrows indicate the relative position and transcriptional direction of genes. Similar processes were applied for deletion of the prophage Phm3.


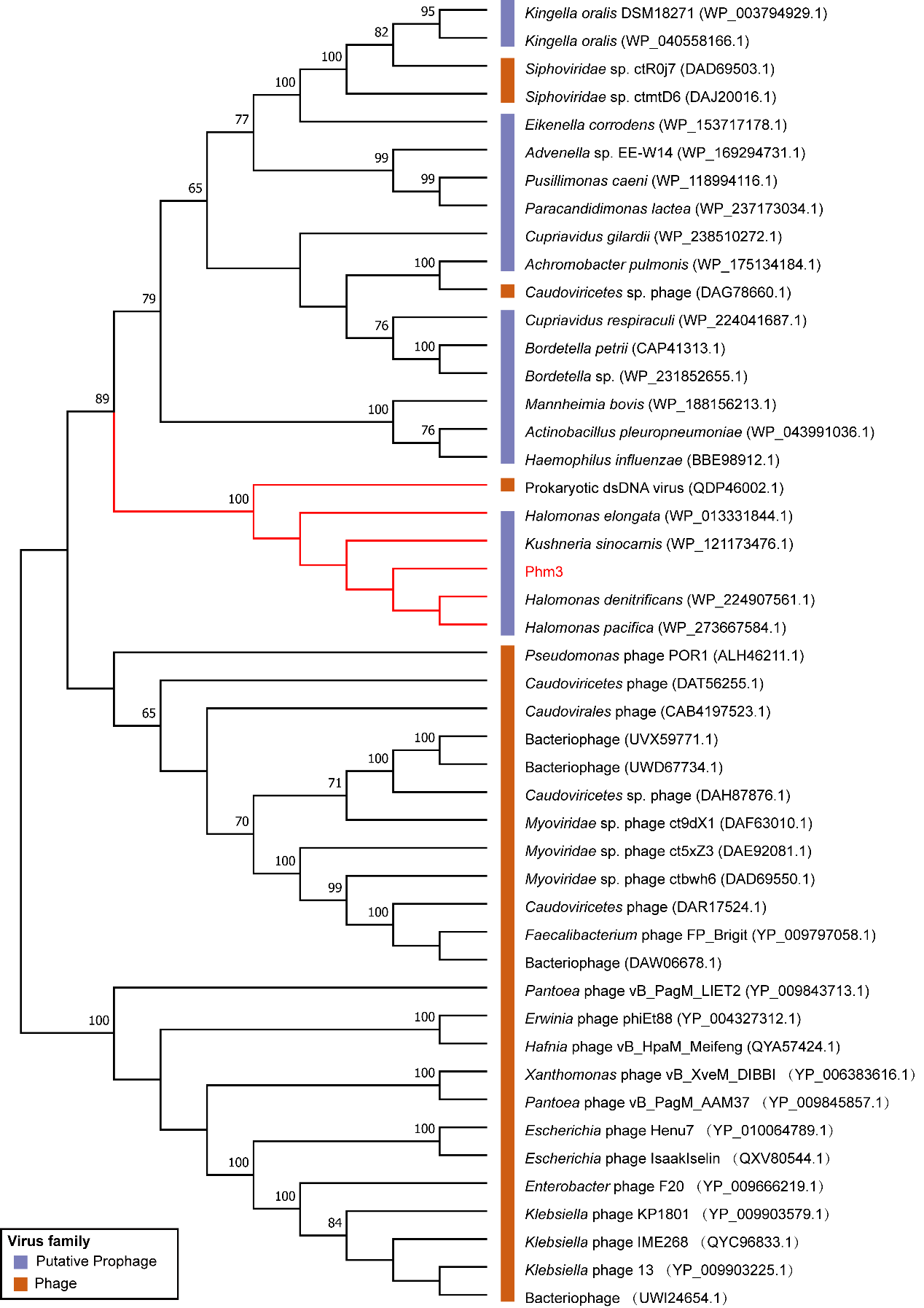


**Figure S3.** **Phylogenetic tree of Phm3 major capsid protein homologues.**

A phylogenetic tree was constructed using MEGA11 with the neighbor-joining method. All the parameters were set to their defaults except for the bootstrap value, which was set to 1,000; the p-distance model was used to calculate the distance; and the gap/missing data treatment cut-off was 50%.


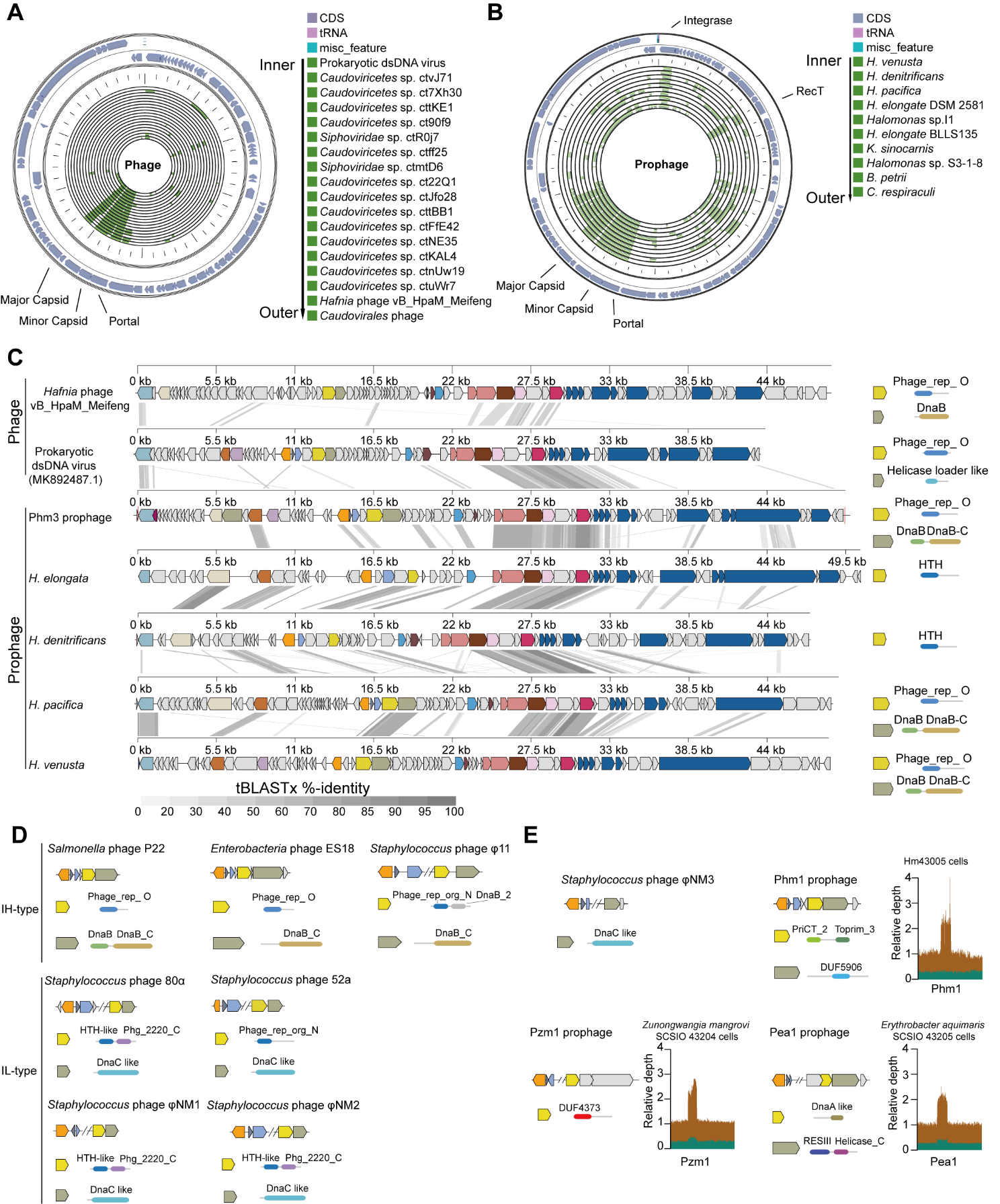


**Figure S4. Comparative genomic analysis of the Phm3 prophage.**

(**A**) The feature rings are shown starting from the outermost ring. 1. Forward strand genes; 2. Reverse strand genes. The next 18 rings show amino acid sequence similarity > 30% (BLASTP) between the selected phages. (**B**) 1. Forward strand genes; 2. Reverse strand genes. The next 10 rings show amino acid sequence similarity > 30% (BLASTP) between other predicted prophages. (**C**) The genetic features of representative phages and prophages were organized and aligned with Phm3 by tBLASTx using Easyfig (version 2.1). The sequence similarity percentage is indicated by the intensity of the grey color. The left and right attachment sites within the host chromosome are indicated by red vertical bars. The arrows represent the relative position and transcriptional direction of genes. (**D**) The replication modules of reported prophages with RPE pathways [2, 3]. (**E**) The replication modules of reported prophages without RPE pathways [2, 3]. The ovals represent protein domain, and different domains are represented by different colors. Conserved domains shown include: Bacteriophage replication protein O: Phage_rep_O (PF04492); DnaB-like helicase N terminal domain: DnaB (PF00772); N-terminal phage replisome organizer: Phage_rep_org_N (PF09681); Replication initiation and membrane attachment: DnaB_2 (PF07261); DnaB-like helicase C terminal domain: DnaB_C (PF03796); Conserved phage C-terminus (Phg_2220_C): Phg_2220_C (PF09524); DUF4373 (PF14297); DUF5906 (PF19263); Helix-turn-helix domain: HTH_36 (PF13730); Type III restriction enzyme, res subunit: ResIII (PF04851); Helicase conserved C-terminal domain: Helicase_C (PF00271); Primase C terminal 2: PriCT-2 (PF08707); Toprim domain: Toprim_3 (PF13362); The DnaC-like (helicase loader), DnaA-like and HTH-like domains were annotated by Foldseek. Phage_rep_org_N is the conserved domain in a recently identified replication initiator.


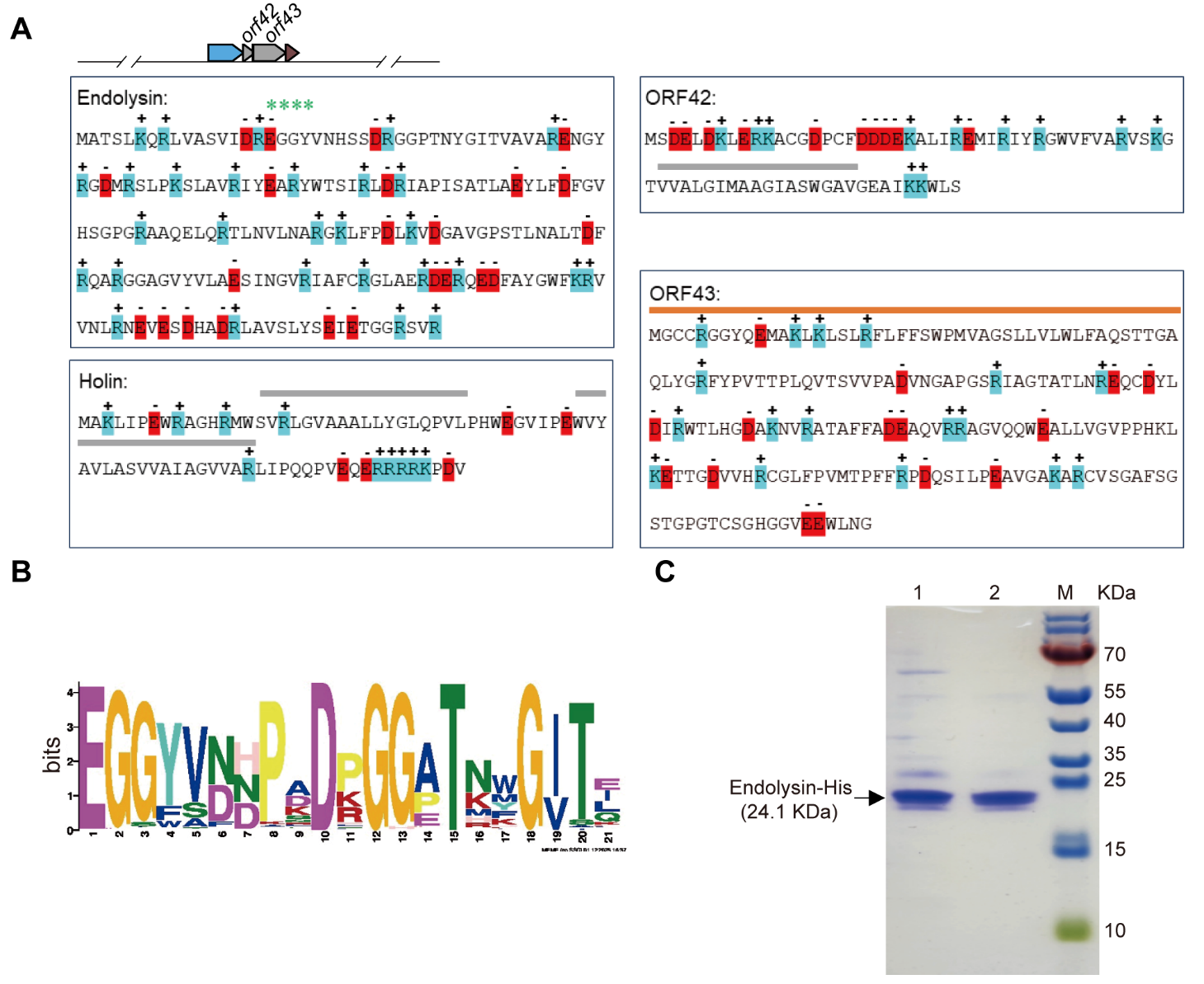


**Figure S5. Lytic module of the Phm3 phage.**

(**A**) Amino acid sequences of all four identified lysis-related proteins and transmembrane domain-containing proteins are shown in the boxes. The predicted transmembrane domains (TMDs) are shown under grey lines. The signal peptide of ORF43 is shown under the orange line. The catalytic residue EGGY motif in endolysin is marked with green asterisks. (**B**) A total of 500 sequences of endolysin homologues were subjected to motif scanning, and the sequence profiles of the conserved motifs are shown. (**C**) SDS‒PAGE of purified endolysin from *E. coli* BL21(DE3). Lanes 1 and 2 show the endolysin eluted with different elution buffers.

**Table S1.** Bacterial strains and plasmids used in this study.

| Bacterial strains and Plasmids | | Description | Source | |
| --- | --- | --- | --- | --- |
| *H.meridiana* SCSIO 43005 | Isolated from Coral samples of *G. fascicularis*. | | | [4] |
| *H.meridiana* SCSIO 43005 Δ*int* | Gene CTT34_*10025*, was knocked out in genome of *H.meridiana* SCSIO 43005. | | | This study |
| *H.meridiana* SCSIO 43005 Δ*rep* | Gene CTT34_*10175*, was knocked out in genome of *H.meridiana* SCSIO 43005. | | | This study |
| ΔPhm1 | All 54 genes from CTT34_*01425* to CTT34_*01695* encoded by Phm1 were knocked out in *H.meridiana* SCSIO 43005 | | | This study |
| ΔPhm3 | All 77 genes from CTT34_*10020* to CTT34_*10405* encoded by Phm3 were knocked out in *H.meridiana* SCSIO 43005 | | | This study |
| ΔPhm1 and Phm3 | All 54 genes from CTT34_*01425* to CTT34_*01695* encoded by Phm1 and 77 genes from CTT34_*10020* to CTT34_*10405* were knocked out in *H.meridiana* SCSIO 43005 | | | This study |
| *H.meridiana* SCSIO 43005 *mcp::gfp* | CTT34_*10290* gene encoding the Phm3 major capsid protein in-frame fused with a *gfp* gene in genome of *H.meridiana* SCSIO 43005 | | | This study |
| *H.meridiana* SCSIO 43005 *mcp::gfp* Δ*endolysin* | Gene *endolysin*, CTT34_*10225*, was knocked out in genome of *H.meridiana* SCSIO 43005 *mcp::gfp* | | | This study |
| *H.meridiana* SCSIO 43005 *mcp::gfp* Δ*holin* | Gene *holin*, CTT34_*10240*, was knocked out in genome of *H.meridiana* SCSIO 43005 *mcp::gfp* | | | This study |
| *H.meridiana* SCSIO 43005 *mcp::gfp* Δ*orf 43* | Gene CTT34_*10235*, was knocked out in genome of *H.meridiana* SCSIO 43005 *mcp::gfp* | | | This study |
| *H.meridiana* SCSIO 43005 *mcp::gfp orf42* amber mutation | Gene CTT34_*10230*, was terminated prematurely in genome of *H.meridiana* SCSIO 43005 *mcp::gfp* | | | This study |
| ***E.coli* Strain** |  | | |  |
| WM3064 | *thrB*1004 *pro* *thi rpsL hsdS lacZ*ΔM15 RP4-360 Δ(*araBAD*)567 Δ*dapA*1341::[*erm pir*] | | | Lab stock |
| BL21 (DE3) | F*-* *ompT hsdS_B_(r_B_^-^m_B_^-^) gal dcm λ*(DE3) Ω P_tacUV5_::T7 *polymerase* | | | Lab stock |
| ER2738 | F´ *proAB* *lacZ*Δ*M15* Tn10 (Tetr)], *fhuA2*, *glnV*, Δ*(lac-proAB)5*, Δ(*hsdSMR*-*mcrB*)*5, thi-1* | | | Lab stock |
| Top10 | F-, *mcrA*Δ(*mrr-hsd RMS-mcrBC*), *φ80*, *lacZ*Δ*M15*, Δ*lacX74*, *recA1*, *ara*Δ*139*Δ*(ara-leu)7697， galU*, *galK*, *rpsL* (SmR) *endA1*, *nupG* | | | Lab stock |
| BTH101 | F-, λ-, *araC14*, *leuB6* (Am), Δ*(gpt-proA)62*, *lacY1*, *glnX44*(AS), *galK2*(Oc), *recA13*, *rpsL20*(StrR), *xyl A5*, *mtl-1*, *thi-1*, [*hsdS20*] | | | Lab stock |

**Table S1.** Bacterial strains and plasmids used in this study. (continued)

| Bacterial strains and Plasmids | Description | Source |
| --- | --- | --- |
|  | **Plasmids** |  |
| pMBL*cas9* | Cm^R^; Expression of Cas9 and target single-guide RNAs (sgRNAs) for prophage deletion | [1] |
| pgRNA-bacteria | Amp^R^; The template plasmid used to amplify the sgRNAs Addgene | Addgene |
| pMBL*cas9*-Phm1 *integrase* | CmR; pMBL*cas9* containing a sgRNA element targeting the *integrase* gene of prophage Phm1 | This study |
| pMBL*cas9*-Phm3 *integrase* | CmR; pMBL*cas9* containing a sgRNA element targeting the *integrase* gene of prophage Phm1 | This study |
| pK18*mobsacB* | Km^R^; A suicide vector for generating in-frame deletions in *H.meridiana* SCSIO 43005 strains | [5] |
| pK18*mobsacB*-*mcp*::*gfp* | Km^R^; pK18*mobsacB* containing the fused gene of *gfp* gene fused with the *mcp* gene, and the homologs arms of *mcp* | This study |
| pK18*mobsacB*-*endolysin* | Km^R^; pK18*mobsacB* containing the homologs arms of Phm3 *endolysin* | This study |
| pK18*mobsacB*-*holin* | Km^R^; pK18*mobsacB* containing the homologs arms of Phm3 *holin* | This study |
| pK18*mobsacB*-*orf43* | Km^R^; pK18*mobsacB* containing the homologs arms of Phm3 *orf43* | This study |
| pK18*mobsacB*-*orf42* | Km^R^; pK18*mobsacB* containing the homologs arms of Phm3 *orf43* and the *orf42* sequence was mutated | This study |
| pHGECm | Cm^R^, IPTG inducible expression vector in *H.meridiana* SCSIO 43005 strains | [6] |
| pHGECm-*endolysin* | Cm^R^; over-expression vector for *endolysin* | This study |
| pHGECm-*orf42* | Cm^R^; over-expression vector for *orf42* | This study |
| pHGECm-*orf43* | Cm^R^; over-expression vector for *orf43* | This study |
| pHGECm-*holin* | Cm^R^; over-expression vector for *holin* | This study |
| pHGECm- *orf43*::*mCherry* | Cm^R^; over-expression vector containing the gene of *mCherry* was fused with the *orf43* gene | This study |
|  |  |  |

**Table S1.** Bacterial strains and plasmids used in this study. (continued)

| Bacterial strains and Plasmids | Description | Source |
| --- | --- | --- |
| pBAD | Amp^R^, Arabinose inducible expression vector in *H.meridiana* SCSIO 43005 strains | [7] |
| pBAD-*gfp*::*orf42* | Cm^R^; over-expression vector containing the gene of *gfp* was fused with the *orf42* gene | This study |
| pET28b | Km^R^, IPTG inducible expression and purified vector | Novagen |
| pET28b-*endolysin*-*his* | Km^R^; pET28b P_T7-lac_::*endolysin* with C-terminal His-tagged | This study |
| pKT25 | Km^R^, encoding T25 fragment | [8] |
| pUT18C | Amp^R^, encoding T18 fragment | [8] |
| pKT25-*orf43* | Km^R^, *orf43* was fused to the C termini of adenylate cyclase in pKT25 | This study |
| pUT18C-*orf42* | Amp^R^, *orf42* was fused to the C termini of adenylate cyclase in pUT18C | This study |
| Cm^R^, Km^R^, Gm^R^, Amp^R^ indicate chloramphenicol, kanamycin, gentamycin, and ampicillin resistance, respectively. | | |

**Table S2.** The primers used in this study.

| Primer /Purpose | Primer Sequence (listed 5’ to 3’) | Description |
| --- | --- | --- |
| Primers used for construction of strain ΔPhm1 | | |
| SgRNA_Phm1_Int_F | AACCAAGGGAGCAGTTACGAGTTTTAGAGCTAGAAATAGCAAGTTAAAATAAGGC | Construction of pMBL*cas9*-Phm1 *integrase* |
| SgRNA_Phm1_Int_R | TCGTAACTGCTCCCTTGGTTACTAGTATTATACCTAGGACTGAGCTAGCTGTCAA |  |
| SgRNA_Ahd I_F | GTAGTTATCTACACGACGGGGAATTCTAAAGATCTTTGACAG |  |
| SgRNA_Phm1_up_R | TCCAGCCGCTGCCCATTGCCCTAGACTCGAGTAAGGATCC |  |
| SgRNA_Phm1_up_F | GGATCCTTACTCGAGTCTAGGGCAATGGGCAGCGGCTGGA |  |
| Phm1_up_down_R | TCAATTCCCGCCGCCTCCACCACTCACTGCCCTTATACGTCCA |  |
| Phm1_up_down_F | TGGACGTATAAGGGCAGTGAGTGGTGGAGGCGGCGGGAATTGA |  |
| Phm1_down_AhdI_R | CATCCATAGTTGCCTGACTCGACTTCACAGTGCCCAAAGC |  |
| Phm1_mcp_F | ATGCCCAAGAGCATTCAAGAAC | Verification of strain ΔPhm1 |
| Phm1_mcp_R | TTAGGAAGCCGCGCACTGGA |  |
| Phm1 attB_F | GGGGGCGACATGGTTTCGAC |  |
| Phm1 attB_R | GTGTCACTTCCTTCTCGATAC |  |
| Primers used for construction of strain ΔPhm3 | | |
| SgRNA_Phm3_Int_F | GTATTGGGATAAAGCAGGGAGTTTTAGAGCTAGAAATAGCAAGTTAAAATAAGGC | Construction of pMBL*cas9*-Phm3 *integrase* |
| SgRNA_Phm3_Int_R | TCCCTGCTTTATCCCAATACACTAGTATTATACCTAGGACTGAGCTAGCTGTCAA |  |
| SgRNA_Ahd I_F | GTAGTTATCTACACGACGGGGAATTCTAAAGATCTTTGACAG |  |
| SgRNA_Phm3_up_R | AGCTGAACGGCTTGGTGAAGCTAGACTCGAGTAAGGATCC |  |
| SgRNA_Phm3_up_F | GGATCCTTACTCGAGTCTAGCTTCACCAAGCCGTTCAGCT |  |
| Phm3_up_down_R | TTGATTGAGGTGTTACGCTTTGGTGCGGATGGGGAGACTC |  |
| Phm3_up_down_F | GAGTCTCCCCATCCGCACCAAAGCGTAACACCTCAATCAA |  |
| Phm3_down_AhdI_R | CATCCATAGTTGCCTGACTCTCCGAGCCATAAATGGCGAT |  |
| phm3_mcp_F | ATGGCTCGGCCTAAGTGG | Verification of strain ΔPhm3 |
| phm3_mcp_R | TCATGTCAGTCTTGCCTCGTC |  |
| Phm3 attB_F | TGCACTAGCTGTGTAATAGCCTC |  |
| Phm3 attB_R | TACTCGACGACGTCCAGCTCGTA |  |

**Table S2.** The primers used in this study. (continued)

| Primer /Purpose | Primer Sequence (listed 5’ to 3’) | Description |
| --- | --- | --- |
| Primers used for construction of strain *H.meridiana* SCSIO 43005 *mcp::gfp* | | |
| Phm3 pk18_up_F BamH I | ATAAGATTAGTCACTGGGTACGTGAATGCCTCGTATGAC | Construction of pK18*mobsacB*-*mcp*::*gfp* |
| Phm3 mcp_R GFP | AGTTCTTCTCCTTTACTAACTCCCACGGCTTTAGTGAGTGAC |  |
| Phm3 mcp_GFP_F | GTCACTCACTAAAGCCGTGGGAGTTAGTAAAGGAGAAGAACT |  |
| Phm3 gfp_R | CAAACTCCCACGGCTTTATTTGTATAGTTCATCC |  |
| Phm3 down_F | GGATGAACTATACAAATAAAGCCGTGGGAGTTTG |  |
| Phm3 pk18_down_R_Hind III | CGGCCAGTGCCAAGCTTCACGTAAGCTCATGGTTGCCA |  |
| mcp::gfp_LF | GTGACCGATGGCGGCGCCGTT | Verification of strain *H.meridiana* SCSIO 43005 *mcp*::*gfp* |
| mcp::gfp_LR | GTGGTGCCAGTCTCGGGGTCA |  |
| mcp::gfp_SF | TGATGCCTACGGCTACAGCCTGGA |  |
| mcp::gfp_SR | GCCAGCACGCCGCGCTTGAGC |  |
| Primers used for construction of strain *H.meridiana* SCSIO 43005 Δ*int* | | |
| Int UP-F | ATAAGATTAGTCACTGGGATCAATGACGGCGAGACGCTGGA | Construction of pK18*mobsacB*- Δ*int* |
| Int UP-R | TCCGCACCAATTTGATTTACCTCACGTCCCAGTGCTTGCTC |  |
| Int Down-F | GAGCAAGCACTGGGACGTGAGGTAAATCAAATTGGTGCGGA |  |
| Int Down-R | CGGCCAGTGCCAAGCTCGGTCGTCACCACATCATCA |  |
| Int-SF | AAGACAACAGGGGAGAAGCG | Verification of strain *H.meridiana* SCSIO 43005 Δ*int* |
| Int-SR | TAGCCACTGCGCTTGATTGA |  |
| Int-LF | CGAGTCGTGCGAGAAGATCA |  |
| Int-LR | CGGATGCGACATAGATGA |  |
| Primers used for construction of strain *H.meridiana* SCSIO 43005 Δ*rep* | | |
| PK18-UP-Rep-F | CATAAGATTAGTCACTGGGGATCCTGTTGGACGAGATGGGCA | Construction of pK18*mobsacB*-Δ*rep* |
| UP-Rep-R | CATCTCGGTATTCCATGCTTATTGAGGGCCCCTTGGTT |  |
| Rep-DN-F | AACCAAGGGGCCCTCAATAAGCATGGAATACCGAGATG |  |
| PK18-Rep-DN-R | CGACGGCCAGTGCCAAGCTTTTCCTTTGAAGCAGCAAG |  |
| LF-Rep | TTTCGTTGCGCAAGTTCTGG | Verification of strain *H.meridiana* SCSIO 43005 Δ*rep* |
| SF-Rep | ATGCACAAAAACGGGAAGGC |  |
| SR-Rep | CCAGGTCATCGATCCAGCTC |  |
| LR-Rep | AAGGATCTTGGCCGACTTGG |  |

**Table S2.** The primers used in this study. (continued)

| Primer /Purpose | Primer Sequence (listed 5’ to 3’) | Description |
| --- | --- | --- |
| Primers used for construction of strain *H.meridiana* SCSIO 43005 *mcp*::*gfp* Δ*endolysin* | | |
| Endolysin UP_F | ATAAGATTAGTCACTGGGATGAACGATAGGAAATCACTACC | Construction of pK18*mobsacB*-*mcp*::*gfp* Δ*endolysin* |
| Endolysin UP_R | GACAGAAGGCGATGCGCATGGACACCTCGGAAATAA |  |
| Endolysin Down_F | TTATTTCCGAGGTGTCCATGCGCATCGCCTTCTGTC |  |
| Endolysin Down_R | CGGCCAGTGCCAAGCTGATTGATCAGGTCGGAAG |  |
| Endolysin_SF | AACGCCCGAAACCGAGTAAT | Verification of strain *H.meridiana* SCSIO 43005 *mcp*::*gfp* Δ*endolysin* |
| Endolysin_SR | CAGCGTCCACCGAATATCCA |  |
| Endolysin_LF | AGTTAAGGCGCTGCAGGATT |  |
| Endolysin_LR | GCTGCAGGCCATAGAGTAGG |  |
| Primers used for construction of strain *H.meridiana* SCSIO 43005 *mcp::gfp* Δ*holin* | | |
| Holin up_F | TCATAAGATTAGTCACTGGGATCACGCCGACCGATTGG | Construction of pK18*mobsacB*-*mcp*::*gfp* Δ*holin* |
| Holin up_R | TCGACAGGCTGCTGCGGGATCATTCGGGGATCAGCTTAGCC |  |
| Holin dn_F | GGCTAAGCTGATCCCCGAATGATCCCGCAGCAGCCTGTCGA |  |
| Holin dn_R | ACGACGGCCAGTGCCAAGCTCATGGGTACCGTTCACCCTT |  |
| Holin_LF | CAACGTGCTGAACGCTCGCGG | Verification of strain *H.meridiana* SCSIO 43005 *mcp*::*gfp* Δ*holin* |
| Holin_SF | TTATCCTGTCACCACACCGC |  |
| Holin_SR | GTGATTCGTTTCTCGCGCTC |  |
| Holin_LR | TTCGCCGTCCTTGGTCTTAC |  |
| Primers used for construction of strain *H.meridiana* SCSIO 43005 *mcp::gfp* Δ*orf43* | | |
| Orf43 up F | TCATAAGATTAGTCACTGGGATCACCGTCGCAGTCGCTCGA | Construction of pK18*mobsacB*-*mcp*::*gfp* Δ*orf43* |
| Orf43 up up R | CGGGAACAGGCCACAGCGGTCAGAGCACGAGCAGTGAGCC |  |
| Orf43 up Down F | GGCTCACTGCTCGTGCTCTGACCGCTGTGGCCTGTTCCCG |  |
| Orf43 up Down R | ACGACGGCCAGTGCCAAGCTAGAATTGCCCGCATATCCAG |  |
| Orf43_LF | GCGATGGTCAAAGCGTGTAC | Verification of strain *H.meridiana* SCSIO 43005 *mcp*::*gfp* Δ*orf43* |
| Orf43_SF | TTGCGGAGTACCTGTTCGAC |  |
| Orf43_SR | GCTGCAGGCCATAGAGTAGG |  |
| Orf43_LR | TTCGCCGTCCTTGGTCTTAC |  |

**Table S2.** The primers used in this study. (continued)

| Primer /Purpose | Primer Sequence (listed 5’ to 3’) | Description |
| --- | --- | --- |
| Primers used for construction of strain *H.meridiana* SCSIO 43005 *mcp::gfp orf42* amber mutaion | | |
| Orf42_Up F（PK18） | TCATAAGATTAGTCACTGGGATCACCGTCGCAGTCGCTCGA | Construction of pK18*mobsacB*-*mcp*::*gfp* Δ*orf42* mutaion |
| Orf42_up R | AAGGGTCACCGCAGGCTTAGCGCTCCAGCTTGTCTAGTT |  |
| Orf42_Down F | AACTAGACAAGCTGGAGCGCTAAGCCTGCGGTGACCCTT |  |
| Orf42_Down R（PK18） | ACGACGGCCAGTGCCAAGCTAGAATTGCCCGCATATCCAG |  |
| Orf42_LF | GCGATGGTCAAAGCGTGTAC | Verification of strain *H.meridiana* SCSIO 43005 *mcp*::*gfp* Δ*orf42* mutation |
| Orf42_SF | TTGCGGAGTACCTGTTCGAC |  |
| Orf42_SR | GCTGCAGGCCATAGAGTAGG |  |
| Orf42_LR | TTCGCCGTCCTTGGTCTTAC |  |
| Primer used for construction of expression vectors | | |
| Edolysin-F | AACAATTTCACACAGGAGAGATGGCTACCAGCCTAAAACA | Construction of pHGECm-*endolysin* |
| Edolysin-R | ATCCGCCAAAACAGCCAAGCTTCATCGGACACTGCGGCC |  |
| Orf42-F | AACAATTTCACACAGGAGAGATGTCCGATGAACTAGACAA | Construction of pHGECm-*orf42* |
| Orf42-R | ATCCGCCAAAACAGCCAAGCTTTAGCTTAGCCATTTCTTG |  |
| Orf43-F | AACAATTTCACACAGGAGAGATGGGGTGCTGTCGGGGAGGCTATCAA | Construction of pHGECm-*orf43* |
| Orf43-R | ATCCGCCAAAACAGCCAAGCTTTAGCCATTCAGCCACTCCTCGA |  |
| Holin-F | AACAATTTCACACAGGAGAGATGGCTAAGCTGATCCCCGAAT | Construction of pHGECm-*holin* |
| Holin-R | ATCCGCCAAAACAGCCAAGCTTCAAACATCGGGCTTCCTC |  |
| Primers for protein expression and purification | | |
| Endolysin-F NcolI | ACTTTAAGAAGGAGATATACATGGCTACCAGCCTAAAACAACGG | Construction of pET28b-*endolysin*-*his* |
| Endolysin-R1 | TTAGTGATGATGATGATGATGTCGGACACTGCGGCCCCCTGTCTCAA |  |
| Endolysin-R2 HindIII | CTCGAGTGCGGCCGCAAGCTTTAGTGATGATGATGATGATG |  |
| Primers for construction of bacterial two-hybrid (BACTH) assays vector | | |
| pUT18c_*orf42*_F | ACTCTAGAGGATCCCCGGGTACCGTCCGATGAACTAGACAAGC | Construction of pUT18C-*orf42* |
| pUT18c_ *orf42*_R | ATTACTTAGTTATATCGATGAATTTTAGCTTAGCCATTTCTTGATAGC |  |
| pKT25_ *orf43*_F | CTAGAGGATCCCCGGGTACCTGGGTGCTGTCGGGGAGGCTAT | Construction of pKT25-*orf43* |
| pKT25_ *orf43*_R | GAATTCTTAGTTACTTAGTCAGCCATTCAGCCACTCCTCGAC |  |

**Table S2.** The primers used in this study. (continued)

| Primer /Purpose | Primer Sequence (listed 5’ to 3’) | Description |
| --- | --- | --- |
| Primers for construction of proteins localization vector | | |
| pBAD_GFP_up_F | TAACAGGAGGAATTAACATGAGTAAAGGAGAAGAACTT | Construction of pBAD-*gfp*::*orf42* |
| pBAD_GFP_up_R | AGCTTGTCTAGTTCATCGGATTTGTATAGTTCATCCATGC |  |
| pBAD_*orf42*_dn_F | GCATGGATGAACTATACAAATCCGATGAACTAGACAAGCT |  |
| pBAD_ *orf42*_dn_R | GTTTTTGTTCTACGTAAGCTTTTAGCTTAGCCATTTCTTGA |  |
| pTac_*orf43*_up_F | CAATTTCACACAGGAGAGAATTCATGGGGTGCTGTCGGGGAGG | Construction of pHGECm- *orf43*::*mCherry* |
| pTac_*orf43*_up_R | TCCTCCTCGCCCTTGCTCACGCCATTCAGCCACTCCTCG |  |
| pTac_mCherry_up_F | CGAGGAGTGGCTGAATGGCGTGAGCAAGGGCGAGGAGGA |  |
| pTac_mCherry_dn_R | ATCCGCCAAAACAGCCAAGCTTTTACTTGTACAGCTCGTCCA |  |
| Primers for qPCR | | |
| Phm1_attB_qF | GACCGCACCGCGTATTAACG | Quantification of *attB* of Phm1 |
| Phm1_attB_qR | CTCGTAAATACCACATCACG |  |
| Phm1_attP_qF | GAGCAGTGTGGTACGCGATGACTC | Quantification of *attP* of Phm1 |
| Phm1_attP_qR | CGTCTCAGATGACAGGGTATTGTC |  |
| Phm2_attB_qF | ACGCGCCAATCATATTGTTC | Quantification of *attB* of Phm2 |
| Phm2_attB_qR | CACTGCCCTATTAACGTTTG |  |
| Phm2_attP_qF | CGTTCATCCATCAAGTGGTC | Quantification of *attP* of Phm2 |
| Phm2_attP_qR | GTATGTTGCCCTCACCTATC |  |
| Phm3_attB_qF | CAGCTCATTCGATTCCAAGG | Quantification of *attB* of Phm3 |
| Phm3_attB_qR | CAACAGCGTGCAGATGCTTG |  |
| Phm3_attP_qF | CAAGCATAGCGCGTGAGACT | Quantification of *attP* of Phm3 |
| Phm3_attP_qR | GGATGATTTGCGGATACACG |  |
| gyrB_qF | AACCCAACGAAGCCAAAGCCATC | Quantification of *gyrB* in SCSIO 43005 |
| gyrB_qR | CGCCGAATCACCCTCCACCAG |  |

**Table S2.** The primers used in this study. (continued)

| Primer /Purpose | Primer Sequence (listed 5’ to 3’) | Description |
| --- | --- | --- |
| Primers for verification of the constructed plasmids | | |
| pHGECm-F | CACCTCGCTAACGGATTCACC | Verification of the constructed plasmids pHGECm |
| pHGECm-R | ACACTACCATCGGCGCTACG |  |
| pET28b-F | TAATACGACTCACTATAGGG | Verification of the constructed plasmids pET28b |
| pET28b-R | TATGCTAGTTATTGCTCAG |  |
| pKT25-F | CGCATCTGTCCAACTTCCGC | Verification of the constructed plasmids pKT25 |
| pKT25-R | CGCCAGGGTTTTCCCAGTCA |  |
| pUT18C-F | GCGAGGGCTATGTCTTCTACG | Verification of the constructed plasmids pUT18C |
| pUT18C-R | GGGCTGGCTTAACTATGCGG |  |
| pBAD-F | ATGCCATAGCATTTTTATCCA | Verification of the constructed plasmids pBAD |
| pBAD-R | TCTGATTTAATCTGTATCAGG |  |

**Table S3.** Annotation of prophages Phm1, Phm2 and Phm3 in Hm43005.

**Table S4.** Transcriptome of prophages Phm1, Phm2 and Phm3 in Hm43005.

**Table S5.** KEGG definition of prophage Phm3 and its flanking sequences.

**Table S6.** Viral genomes were retrieved from the NCBI Viruses database for phylogenetic using Phm3 MCP sequence BlastP.

**Table S7.** Phm3-like MCP obtained from NCBI database for phylogenetic using Phm3 MCP sequence BlastP.

**References**

1. Liu X, Tang K, Zhang D, Li Y, Liu Z, Yao J, et al. Symbiosis of a P2-family phage and deep-sea *Shewanella putrefaciens.* *Environ Microbiol* 2019;**21**:4212-32.

2. Chen J, Quiles-Puchalt N, Chiang YN, Bacigalupe R, Fillol-Salom A, Chee MSJ, et al. Genome hypermobility by lateral transduction*.* *Science* 2018;**362**:207-12.

3. Fillol-Salom A, Bacigalupe R, Humphrey S, Chiang YN, Chen J, Penadés JR. Lateral transduction is inherent to the life cycle of the archetypical *Salmonella* phage P22*.* *Nat Commun* 2021;**12**:6510.

4. Tang K, Zhan W, Zhou Y, Xu T, Chen X, Wang W, et al. Antagonism between coral pathogen *Vibrio coralliilyticus* and other bacteria in the gastric cavity of scleractinian coral *Galaxea fascicularis.* *Sci China Earth Sci* 2020;**63**:157-66.

5. Schäfer A, Tauch A, Jäger W, Kalinowski J, Thierbach G, Pühler A. Small mobilizable multi-purpose cloning vectors derived from the *Escherichia coli* plasmids pK18 and pK19: selection of defined deletions in the chromosome of *Corynebacterium glutamicum.* *Gene* 1994;**145**:69-73.

6. Wang P, Zeng Z, Wang W, Wen Z, Li J, Wang X. Dissemination and loss of a biofilm-related genomic island in marine *Pseudoalteromonas* mediated by integrative and conjugative elements*.* *Environ Microbiol* 2017;**19**:4620-37.

7. Guzman LM, Belin D, Carson MJ, Beckwith J. Tight regulation, modulation, and high-level expression by vectors containing the arabinose PBAD promoter*.* *J Bacteriol* 1995;**177**:4121-30.

8. Karimova G, Pidoux J, Ullmann A, Ladant D. A bacterial two-hybrid system based on a reconstituted signal transduction pathway*.* *Proc Natl Acad Sci U S A* 1998;**95**:5752-6.
